# Frequency of a natural truncated allele of *MdMLO19* in the germplasm of *Malus domestica*

**DOI:** 10.1007/s11032-016-0610-8

**Published:** 2017-01-04

**Authors:** Stefano Pessina, Luisa Palmieri, Luca Bianco, Jennifer Gassmann, Eric van de Weg, Richard G. F. Visser, Pierluigi Magnago, Henk J. Schouten, Yuling Bai, R. Riccardo Velasco, Mickael Malnoy

**Affiliations:** 1 Research and Innovation Centre, Fondazione Edmund Mach, Via Edmund Mach 1, 38010 San Michele all’Adige, Italy; 2 Wageningen UR Plant Breeding, Wageningen University and Research, P.O. Box 386, 6700 AJ Wageningen, the Netherlands; 3 Agroscope, Institute for Plant Production Sciences IPS, Schloss 1, 8820 Wädenswil, Switzerland

**Keywords:** *MLO*, *MdMLO19*, *Malus domestica*, Apple, SNP, Powdery mildew

## Abstract

*Podosphaera leucotricha* is the causal agent of powdery mildew (PM) in apple. To reduce the amount of fungicides required to control this pathogen, the development of resistant apple cultivars should become a priority. Resistance to PM was achieved in various crops by knocking out specific members of the *MLO* gene family that are responsible for PM susceptibility (S-genes). In apple, the knockdown of *MdMLO19* resulted in PM resistance. However, since gene silencing technologies such as RNAi are perceived unfavorably in Europe, a different approach that exploits this type of resistance is needed. This work evaluates the presence of non-functional naturally occurring alleles of *MdMLO19* in apple germplasm. The screening of the re-sequencing data of 63 apple individuals led to the identification of 627 single nucleotide polymorphisms (SNPs) in five *MLO* genes (*MdMLO5, MdMLO7, MdMLO11, MdMLO18*, and *MdMLO19*), 127 of which were located in exons. The T-1201 insertion of a single nucleotide in *MdMLO19* caused the formation of an early stop codon, resulting in a truncated protein lacking 185 amino acids, including the calmodulin-binding domain. The presence of the insertion was evaluated in 115 individuals. It was heterozygous in 64 and homozygous in 25. Twelve of the 25 individuals carrying the insertion in homozygosity were susceptible to PM. After barley, pea, cucumber, and tomato, apple would be the fifth species for which a natural non-functional *mlo* allele has been found.

**Electronic supplementary material:**

The online version of this article (doi:10.1007/s11032-016-0610-8) contains supplementary material, which is available to authorized users.

## Introduction

Powdery mildew (PM) is a relevant disease of apple that, in the absence of chemical control, can reduce yield up to 50% (Yoder 2000). The disease is caused by the obligate biotroph fungus *Podosphaera leucotricha*, and it occurs in all major apple-growing regions of the world (Turechek et al. 2004). Leaves are the most susceptible organ, particularly during the first days after opening, but blossom infections, although less common, are extremely severe because they result in small and stunted fruits, or in no fruit at all (Turechek et al. 2004).

PM is a serious problem for thousands of plant species (Glawe 2008). Luckily, a source of durable resistance exists, which can be achieved by the knockout or knockdown of specific member(s) of the *MLO* gene family, as previously shown by Pavan et al. (2010), Wang et al. (2014), and Pessina et al. (2016a, 2016b). The *MLO* gene family comprises a variable number of members, grouped in seven clades (Acevedo-Garcia et al. 2014; Pessina et al. 2014). *MLO* genes for PM susceptibility (*MLO* S-genes) belong to clade IV, which contains monocot S-genes (Panstruga 2005; Reinstädler et al. 2010; Wang et al. 2014), and clade V, which contains dicot S-genes (Consonni et al. 2006; Bai et al. 2008; Feechan et al. 2008; Winterhagen et al. 2008). Loss of function in *MLO* S-genes leads to PM resistance as demonstrated in barley (Jørgensen 1992), *Arabidopsis thaliana* (Consonni et al. 2006), tomato (Bai et al. 2008), pea (Pavan et al. 2011), wheat (Wang et al. 2014), and cucumber (Berg et al. 2015). It is possible to identify *MLO* S-genes through gene expression analysis: at early stages of PM infection, specific *MLO* S-genes have their expression increased. This was documented in barley (Piffanelli et al. 2002), tomato (Bai et al. 2008), grape (Feechan et al. 2008; Winterhagen et al. 2008), pepper (Zheng et al. 2013), and apple (Pessina et al. 2014). Of the four *MLO* apple genes of clade V*, MdMLO11* and *MdMLO19* are up-regulated during PM infection, whereas *MdMLO5* and *MdMLO7* are not (Pessina et al. 2014). *MdMLO18*, a gene of clade VII, is also responsive to PM infection. Among these PM-inducible apple genes, only *MdMLO19* can be considered an S-gene because its knockdown reduced PM infection up to 75%, whereas the knockdown of *MdMLO11* did not support any reduction of PM infection. A role of *MdMLO18* does not seem likely on the basis of the result of the complementation of resistance test carried out in *A. thaliana* (Pessina et al. 2016a).

Gene silencing technologies, such as RNAi, are currently not accepted by the large majority of the European public (Einsele 2007); accordingly, the EU has the strictest regulation in the world on GMOs (Davison 2010). Therefore, we searched for non-functional alleles of the apple *MLO* S-gene *MdMLO19*, using the natural genetic diversity of apple to develop PM-resistant varieties. The diversity of other four apple *MLO* genes (*MdMLO5, 7, 11*, and *18*) was also studied because they are either members of clade V (*MdMLO5* and *7*), are up-regulated upon PM infection (*MdMLO18*), or both (*MdMLO11)*. In apple, the FruitBreedomics project (http://www.fruitbreedomics.com) opened interesting possibilities making available 63 re-sequenced *Malus domestica* individuals representing the genetic diversity present in the apple germplasm (Bianco et al. 2016). Here, we report on the screening of the 63 re-sequenced genomes, searching for non-functional alleles of five *MLO* genes, i.e., the four members of clade V and *MdMLO18*. Among them, *MdMLO19* is the main gene of interest, but since recent evidences suggested that also *MLO* genes that do not show higher transcription levels after PM inoculation may have a role in PM pathogenesis (Pessina et al. 2016b), *MdMLO5* and *7* were also considered. Furthermore, the evidences of the lack of a role for *MdMLO18* in PM pathogenesis are not final, so it was included in the present study as well. We focused on the mutations located in the exons for simplicity, as their effects can be predicted more easily compared to mutations locating in introns, promoter, and terminator. A non-functional natural allele of *MdMLO19* was found and the link to PM resistance investigated by the genotyping and phenotyping of cultivars, breeding selections and wild species. The possibility of using this allele to introgress durable resistance in apple varieties is discussed as well.

## Materials and methods

### FruitBreedomics re-sequencing data analysis

The genomic regions hosting genes *MdMLO5, MdMLO7, MdMLO11, MdMLO18*, and *MdMLO19* (Pessina et al. 2014) were screened in 63 individuals for which re-sequencing data were available from the design of a 20K and a 480K single nucleotide polymorphism (SNP) array (Bianco et al. 2014, 2016). Only the open reading frames (ORF) of the five genes were considered, whereas the sequences of the promoters and terminators were not screened. For these 63 individuals, SNPs were retrieved from the variant calling format (.vcf) file used for the development of the 480K array (Bianco et al. 2016). A custom bioinformatic script was then written to retrieve all polymorphic sites of just the five genes. Data were stored in a tab-separated value file (.tsv) for further processing. The retrieved SNPs were divided in two groups, depending on if they were located in the exons or in the introns, and only those located in the exons were considered for further analyses. SNP-based nucleotide sequences were deduced, as well as gene-encoded amino acids (aa) sequences, using EMBOSS transeq (http://www.ebi.ac.uk/Tools/st/emboss_transeq/). Mutations were grouped in seven categories: silent substitutions (no aa changes), conservative substitutions (aa substituted with one of similar chemical and sterical properties), semi-conservative substitutions (substitution with an aa with similar sterical properties), non-conservative substitutions (substitution with an aa with different properties), insertions (insertion of one or more aa), deletions (removal of one or more aa), and nonsense mutations (formation of an early stop codon).

### Selection of individuals

In order to study the frequency of the mutations found in the FruitBreedomics dataset and their possible association with PM resistance/susceptibility, 115 individuals from three locations were selected. Phenotypic data from different sources were available for 100 of the individuals considered. Since phenotypic data were the result of different assessment methods, they were analyzed independently.

#### Fondazione Edmund Mach

Two groups of individuals were collected from the orchard of Fondazione Edmund Mach (Italy). The 60 individuals of the first group were collected because their level of resistance was known from the data provided by Mr. Ted L. Swensen (Table S1): very resistant, resistant, susceptible, and very susceptible (Table S2—FEM). The second group of 35 individuals included 10 accessions of wild *Malus* species and 25 cultivars that are commonly used in breeding, commercially relevant or selected because their level of PM resistance/susceptibility was known from direct observation carried out during the years by the breeders of FEM (Table S2—FEM2).

#### Wädenswil

An orchard including 628 apple accessions each represented by 2 tree individuals, located at Agroscope in Wädenswil (Switzerland), was evaluated yearly for 4 years after being left completely untreated with fungicides. PM symptoms were scored every spring using a scale from 1 to 9 (1, complete absence of symptoms; 9, tree completely affected). Eleven individuals were selected among those with the lowest standard deviation between replicates and years (Table S2—Wädenswil).

#### FruitBreedomics

The FruitBreedomics project provided the DNA and the phenotypic information of 10 individuals. Five of them were susceptible to PM, whereas the phenotype of the other five was unknown (Table S2—FruitBreedomics). These latter five were included with the purpose of validating the FruitBreedomics re-sequencing dataset, as they were among the 63 cultivars constituting the said dataset.

### DNA extraction

Leaf samples were ground in liquid nitrogen and DNA was extracted with illustra Nucleon PhytoPure Kit (GE Healthcare, Buckinghamshire, UK). Resulting DNA was quantified with NanoDrop (Thermo Fisher Scientific, Waltham, USA).

### Genotyping by Sanger sequencing

To validate the presence of the insertion of a T at position 1201 in *MdMLO19,* and to genotype a larger set of individuals*,* a 186-bp region was amplified (Fw, 5′-GCATCTTGTCCTCGTATGTAGAATG-3′; Rv, 5′-CGACATCTTCCAACTTCTCATGG-3′) with GoTaq Green (Promega, Fitchburg, USA) and sequenced twice from both ends (Table S2). Sequences were aligned using the Staden package software (Staden 1996).

Sanger sequencing can be easily used to detect homozygous mutations. Conversely, heterozygous mutations are not as obvious. The sequencing electropherogram was expected to show two overlapping peaks in the site of the mutation, one consisting in the wild-type sequence and one in the mutated sequence. However, overlapping peaks might also be the result of sequencing artifact/errors. To rule out this possibility, the 186-bp fragment from the heterozygous cultivar Durello di Forlì was cloned into the gateway vector pENTR/SD-TOPO (Thermo Fisher Scientific, Waltham, USA) and inserted into *Escherichia coli*, which was plated on a selective media. Eight colonies were picked, the plasmids extracted with QIAprep Spin Miniprep kit (Qiagen, Venlo, the Netherland) and sequenced using Sanger technology.

### Canonical correspondence analysis

Canonical correspondence analysis (CCA) as embedded in the PAST software v. 2.17c (Hammer et al. 2001) was performed to determine the relative importance of resistance levels in the spatial organization of genetic diversity among individuals. This analysis, designed to relate species composition to different predictive variables (Ter Braak 1986), has been successfully used to describe relationships between environmental or phenotypical variables and genetic composition (Angers et al. 1999; Dell’Acqua et al. 2014; Zoratti et al. 2015). The analysis was based on a disease levels/genotype matrix. Sanger sequencing was used to assess the genotype of the individuals regarding insertion T-1201.

## Results

### Presence of SNPs in the target *MLO* genes

The screening of the re-sequencing data returned 678 SNPs in the ORF of five *MLO* genes (Table S3), i.e., the four members of clade V and *MdMLO18.* One hundred twenty-seven of the SNPs were located in exons (Table S4). The *MLO* gene with the highest number of SNPs located in exons was *MdMLO19* with 48 SNPs; the gene with the lowest number was *MdMLO5* with 6 (Table S4).

**Fig. 1:**
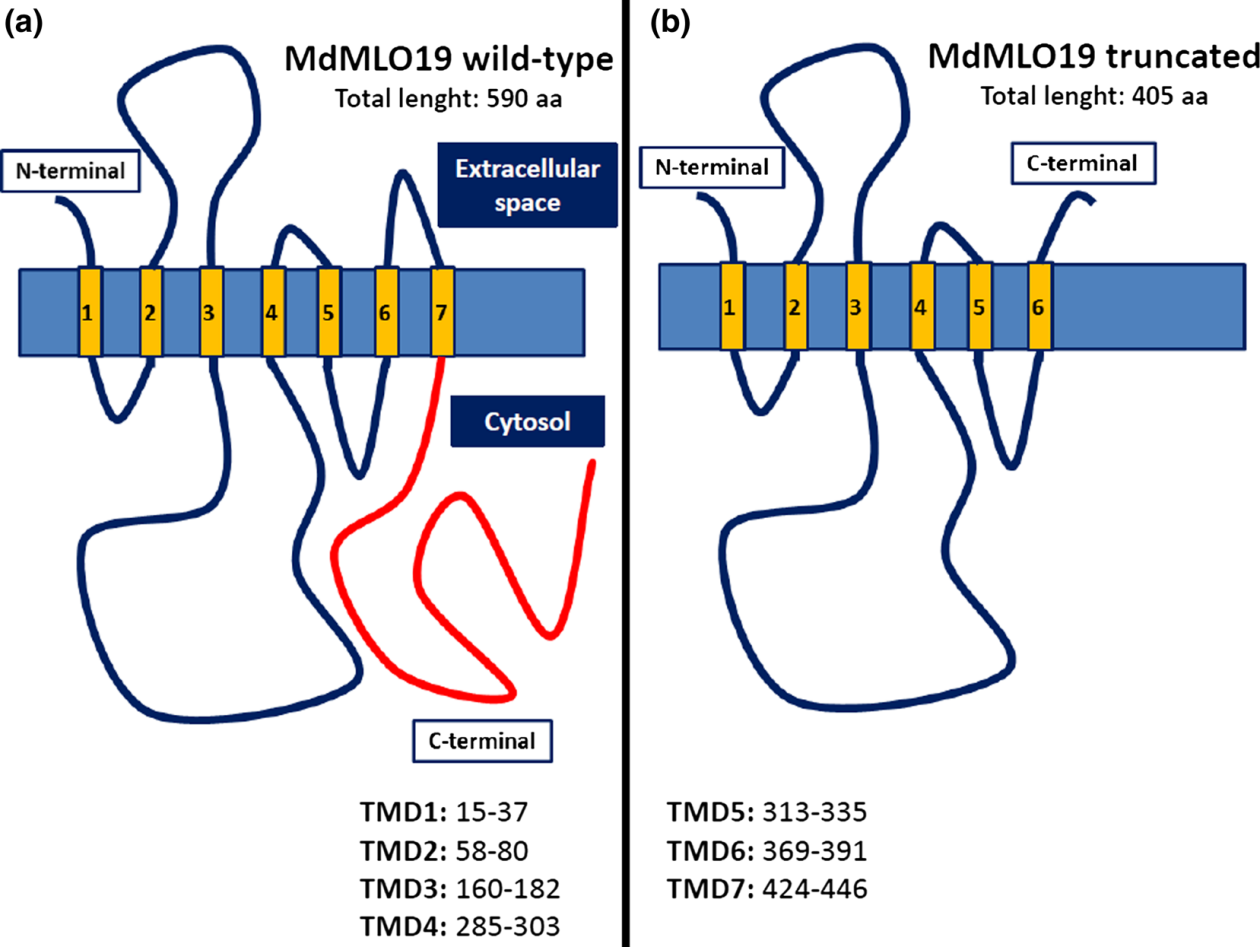
Structures of wild-type (**a**) and truncated (**b**) MdMLO19 proteins. The trans-membrane domains (TMD) are indicated in *yellow*. The wild-type MdMLO19 contains at the C-terminal a calmodulin-binding domain (color figure online)

Sixty-one out of the 127 exon-located SNPs caused silent mutations, and another 30 and 9 caused conservative and semi-conservative substitutions, respectively (conservative: substitution of an aa with one of similar chemical properties; semi-conservative: substitution of an aa with one of similar steric conformation). Twenty-two mutations were non-conservative (Table 1) plus two insertions, two deletions, and a nonsense mutation. One insertion was located at the very end of *MdMLO7,* in position 1676–1680, causing a frameshift that changed the last three amino acids of the protein. The other insertion, T-1201, was located in *MdMLO19* and caused a frameshift of one nucleotide with the formation of an early stop codon (Table 1). The resulting protein would be 405 amino acids long, instead of 590, and would lack both the trans-membrane (TMD-7) and calmodulin-binding domains at the C-terminal (Fig. 1). According to re-sequencing FruitBreedomics data, insertion T-1201 was present in 12 of the 63 genotyped individuals. In six of them, it was homozygous (“Busiard,” “Patte de Loup,” “McIntosh,” “Pepino Jaune,” “Young America,” and “Kronprins”), in the other six heterozygous (“Mela Rozza,” “Priscilla,” “Abbondanza,” “Jonathan,” “Alfred Jolibois,” and “Filippa”). One of the two deletions, G-1181, was remarkable: it was found in *MdMLO19*, where it would cause the formation of an early stop codon. However, this G-1181 deletion was present only in “Pepino Jaune”, where insertion T-1201 was also present in homozygosity. The combination of deletion G-1181 and insertion T-1201 would cause the substitution of five amino acids, but no early stop codon. Since “Pepino Jaune” is homozygous for insertion T-1201 and heterozygous for deletion G-1181, only one of its alleles actually carries insertion T-1201 alone. For this reason, “Pepino Jaune” was included in the genotypes heterozygous for the insertion. The other nonsense mutation found in MdMLO19 was substitution G-1176-A*,* which caused the substitution of a tryptophan with an early stop codon. This SNP was found in “Ajmi”.

**Table 1:** Type of mutations for the 127 SNPs located in introns

	No. of SNPs (exons)	Silent	Conservative	Semi-conservative	Non-conservative	Nonsense	Insertions	Deletions
*MdMLO5*	6	0	4	0	2	0	0	0
*MdMLO7*	24	10	9	2	2	0	1	0
*MdMLO11*	23	9	5	3	6	0	0	0
*MdMLO18*	26	12	6	1	7	0	0	0
*MdMLO19*	48	30	6	3	5	1	1	2
Total	127	61	30	9	22	1	2	2

Insertion T-1201 and nonsense mutation G-1176-A, both located in *MdMLO19,* were selected for further analysis.

### Validation of the presence of insertion T-1201 in the *MdMLO19* gene

**Fig. 2:**
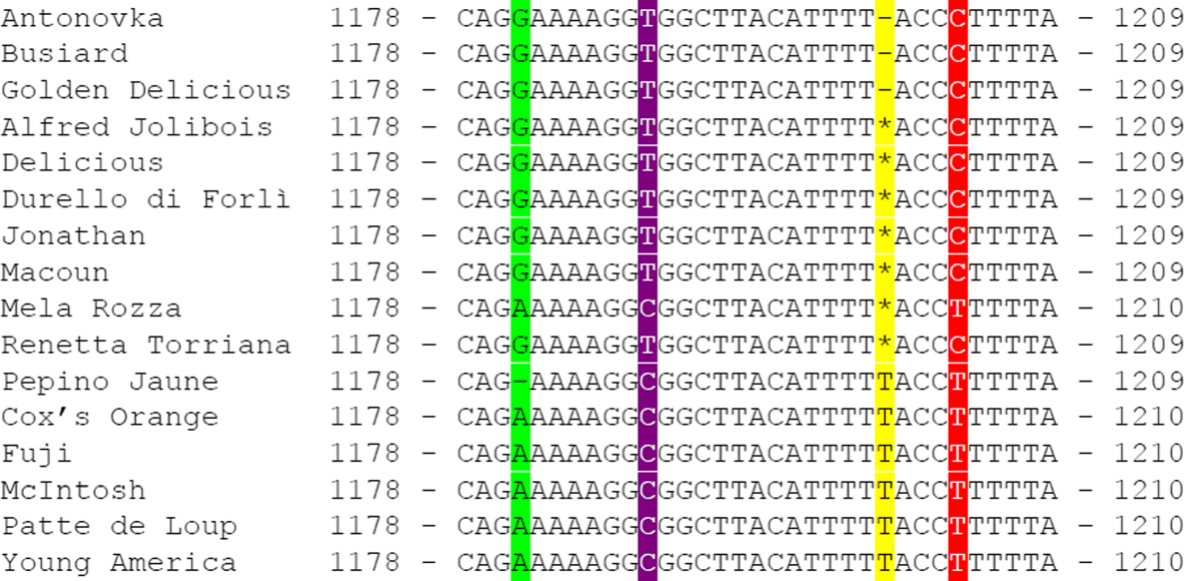
Sequences of a fragment of *MdMLO19* obtained by Sanger sequencing of seven apple individuals. *Colored columns* correspond to SNPs present in the FruitBreedomics re-sequencing dataset and confirmed by Sanger. The *yellow column* highlights position 1201. The *dashes* in the *yellow column* indicates the lack of insertion T-1201, whereas the *asterisks* indicate heterozygosity of the insertion in that individual. The *green*, *purple*, and *red columns* highlight the positions of the three SNPs associated to insertion T-1201 (color figure online)

Sanger sequencing of a fragment of *MdMLO19* in 16 individuals included in the FruitBreedomics re-sequencing dataset showed that 13 of them had insertion T-1201 (Fig. 2). For eight individuals, the electropherograms showed an overlapping of the peaks for A and T in position 1201, suggesting that the insertion was heterozygous (Fig. 2). To rule out the possibility that these overlapping peaks were the result of sequencing artifact/errors, an additional validation was carried out: a fragment of *MdMLO19* from the heterozygous individual “Durello di Forlì” was cloned in a plasmid and sequenced. Of the eight *E. coli* colonies sequenced, four carried insertion T-1201, whereas the other four did not carry it (Fig. S2), indicating that Sanger sequencing is adequate to distinguish heterozygous mutations from homozygous ones.

Sanger sequencing also confirmed the presence of the G-1181 deletion in “Pepino Jaune”, supporting the FruitBreedomics data (Fig. 2), whereas the sequencing of “Ajmi” did not confirm the presence of the nonsense mutation in this individual. No further analysis were carried out on substitution G-1176-A.

The results obtained by Sanger sequencing for insertion T-1201 were compared to those of the FruitBreedomics re-sequencing dataset and found to be conflicting in seven cases (Table S5). The results of Sanger sequencing have been used for further steps of the work.

### Pedigree of apple individuals

Parentages were known for 79 of the 115 individuals considered (Table S2). Two inconsistencies were noted for the Sanger data: “Telamon” did not show the insertion, but one of its parents (“McIntosh”) had it in homozygosity, whereas the other parent (“Golden Delicious”) lacked the insertion. Therefore, “Telamon” should be heterozygous. The same is true for “James Grieve” and its parent “Cox’s Orange Pippin”, as “James Grieve” is homozygous for the absence of the insertion and “Cox’s Orange Pippin” for the presence. The Sanger sequencing confirmed the genetic state of each of these four individuals; therefore, the discrepancies must have other explanations. Possibly, the DNA samples of “Telamon” and “James Grieve” were not true to type.

### Frequency of insertion T-1201 and association with the phenotype

**Fig. 3:**
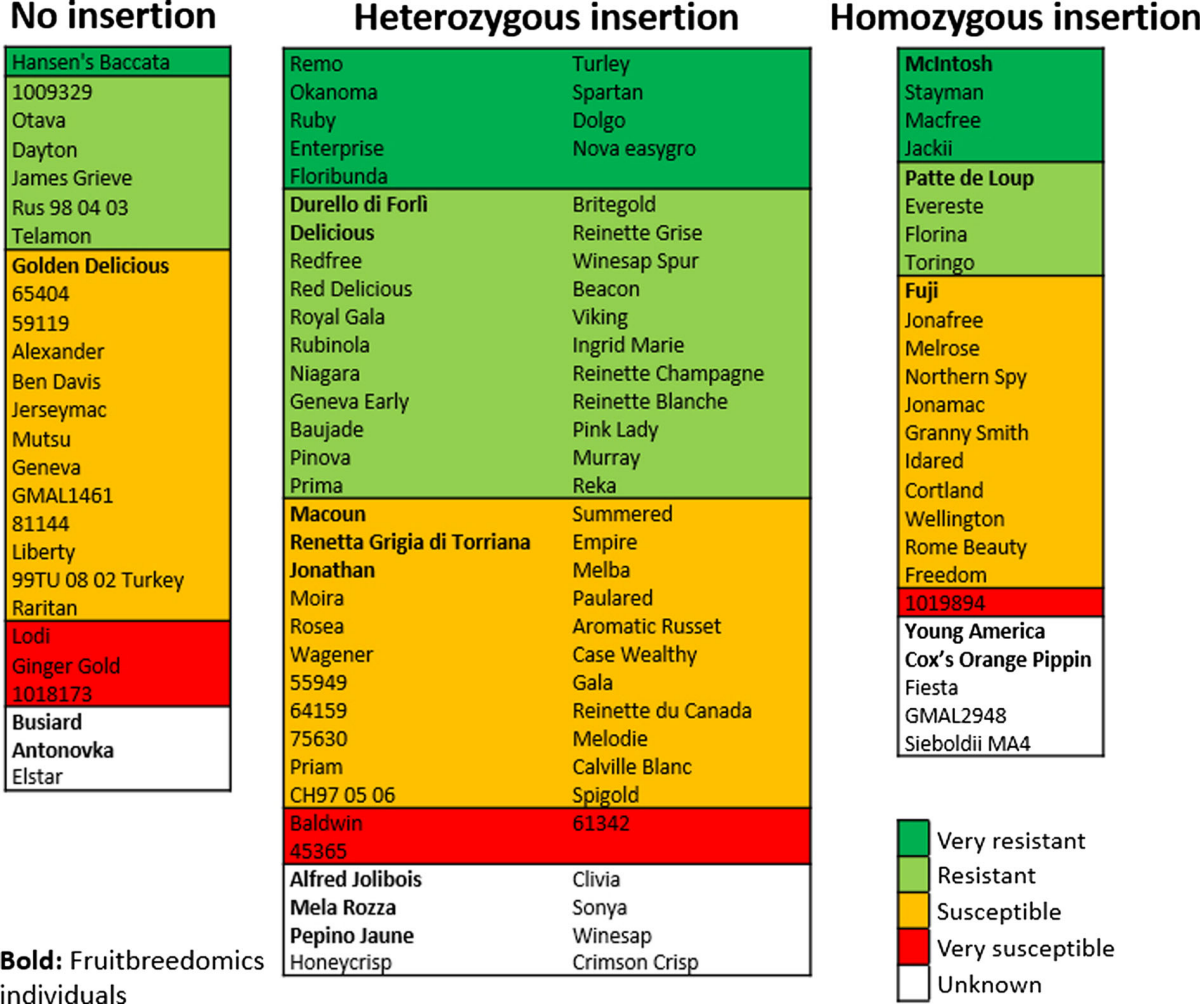
List of apple individuals characterized by the presence or absence of insertion T-1201. The *background color* indicates the level of resistance/susceptibility. The individuals from FruitBreedomics dataset are in *bold*

A 186-bp fragment of *MdMLO19* containing insertion T-1201 was sequenced by Sanger in 115 individuals. The insertion was present in 89 of them, heterozygous in 64, and homozygous in 25 (Fig. 3 and Table S2). The sequencing also showed that, contrary to expectations, 12 of the 25 individuals homozygous for insertion T-1201 were susceptible or very susceptible to PM. Among the individuals considered, there were also three mutants, namely “Royal Gala” (mutant of “Gala”), “Red Delicious” (mutants of “Delicious”), and “Turley Winesap” (mutant of “Winesap”). All of them were identical to their individual of origin (Fig. 3 and Table S2).

**Fig. 4:**
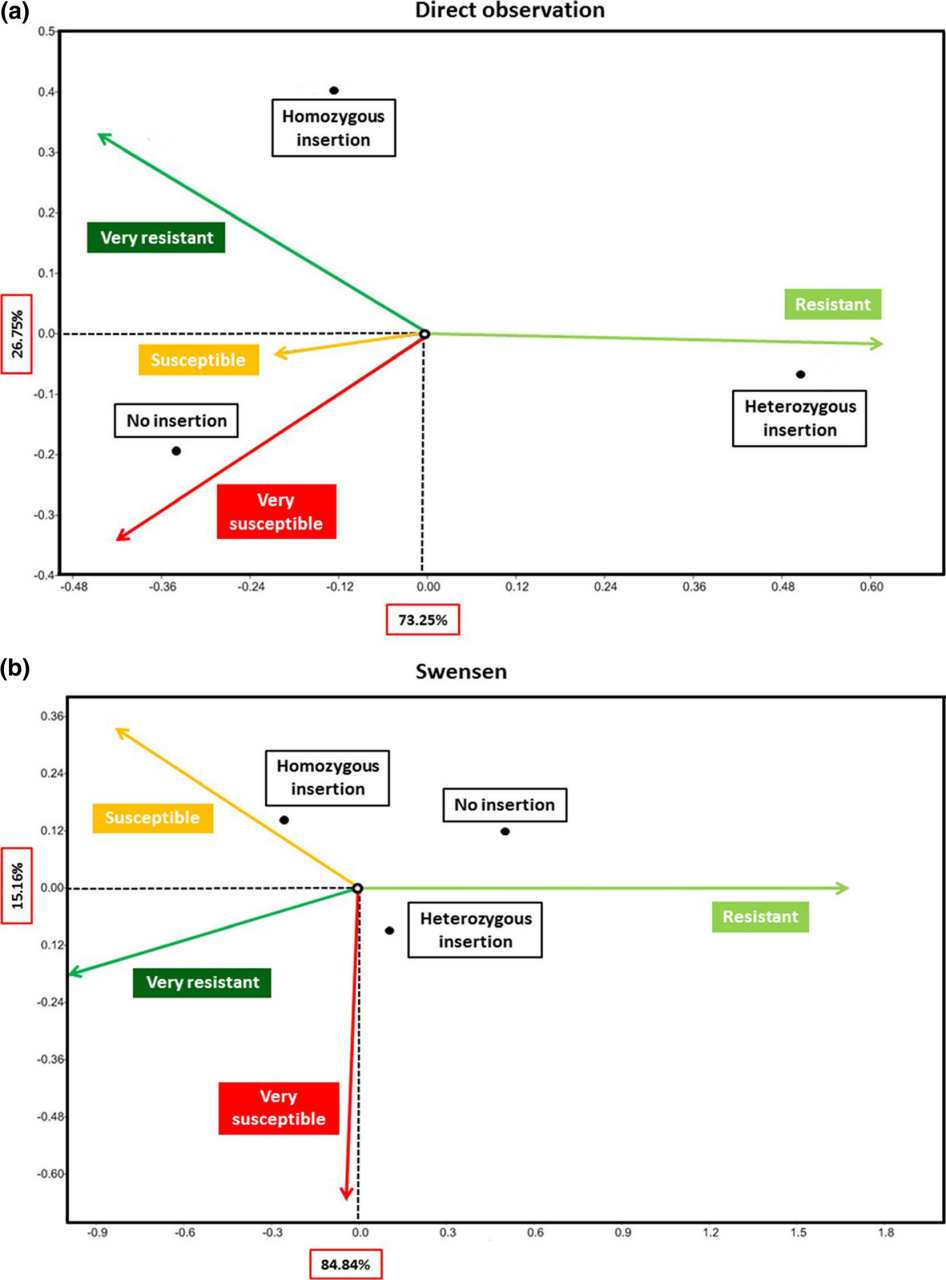
Canonical correspondence analysis (CCA) ordination biplot representing individuals’ aggregation and phenotypical variables. The *arrows* emerging from the origins of the two axes represent the phenotypes, and their position indicates the association with the genotype: the closer the *arrow* is to the *dot* indicating the genetic composition, the stronger is the association. The three genetic compositions in exams are no insertion, heterozygous insertion, and homozygous insertion (*colored boxes*). The four phenotypes considered are very resistant, resistant, susceptible, and very susceptible (*solid arrows*). **a** CCA performed on 23 individuals, the phenotype of which was directly observed by apple breeders in FEM orchard. **b** CCA performed on 60 individuals, the phenotype of which was retrieved from the data provided by Mr. Ted L. Swensen

To analyze the association between the presence/absence of insertion T-1201 and resistance or susceptibility to PM, a subset of the 115 individuals was chosen. Fifteen individuals with no phenotypic data available were excluded, as well as the mutants previously mentioned. Furthermore, the individuals from Wädenswil and from the FruitBreedomics project Table (S2) were not considered for the CCA because their small number did not allow to perform the analysis. Two independent CCAs were carried out for two groups of individuals, the phenotypic data of which were obtained from different sources: data from direct observation (23 individuals) and data provided by Mr. Swensen (60 individuals). To read correctly the CCA biplots showed in Fig. 4, it is important to note that the two axes *x* and *y* have different importance in explaining the significance of the association: for both Fig. 4a, b, the majority of the significance is explained by the *x*-axis (73.25 and 84.84%, respectively). This means that the distance on the x-axis between the points indicating the genotype and the arrows indicating the phenotype is more relevant than the distance on the *y*-axis. Thus, the CCA carried out on the data coming from direct observation (Fig. 4a) showed two associations, one between the very susceptible phenotype and no insertion and the other between resistance and heterozygous insertion. Two partial associations were also noted between high resistance and homozygous insertion and between susceptibility and absence of the insertion. Conversely, the CCA performed on Swensen data (Fig. 4b) did not show any clear association.

## Discussion

The screening of the FruitBreedomics re-sequencing dataset returned 678 SNPs in five *MLO* genes. Not surprisingly, SNP distribution was not balanced between introns and exons: the fewer SNPs in the exons can be explained by positive selection against detrimental mutations, whereas introns mutations are to a large extent neutral and subjected to random fixation (Kimura 1977). The same holds for the predominance of silent and conservative mutations in exons. None of the 127 SNPs found in exons affected any of the 30 amino acids identified by Elliott et al. (2005) as fundamental for the S-genes activity of MLO proteins.

The case of *MdMLO5* deserves a comment: only six SNPs were detected in exons, suggesting that the gene is under intense stabilizing selection. Since this gene is unrelated to the infection caused by *P. leucotricha* (Pessina et al. 2014), PM epidemics should not favor the fixation of new mutations. The opposite situation was observed for *MdMLO19*, the gene with the highest number of SNPs and the only one where nonsense mutations were present, a situation indicating that selection should have favored the fixation of mutations. Two factors may have contributed: first, *MdMLO19* is the primary target of *P. leucotricha,* suggesting a co-evolution of host and pathogen. This means that *MdMLO19* causes susceptibility to PM in apple, and this is a well-known case where gene silencing results in recessive resistance to the pathogen (Pessina et al. 2016a). The second factor is that *MdMLO11*, due to the possible redundancy of its metabolic activity (Pessina et al. 2014), may support a loss of function of *MdMLO19* without drastically reducing plant fitness.

The insertion of a thymine in position 1201 of *MdMLO19* caused a frameshift mutation resulting in an early stop codon located 15–17 bp downstream of the insertion. As a result, the T-1201 insertion causes the translation of a 405 aa protein instead of the 590 aa of the regular protein (Fig. 2). The loss of 185 aa alone would probably compromise the function of MdMLO19; moreover, the C-terminal MLO region carries a calmodulin-binding domain which absence reduces by 50% the capacity of MLO to support infection (Kim et al. 2002). It is reasonable to assume that the truncated MdMLO19 is a non-functional or partially functional protein. Considering that the knockdown of *MdMLO19* resulted in PM resistance (Pessina et al. 2016a), the homozygosity of insertion T-1201 was expected to support PM resistance.

The main purpose of our study was the analysis of the frequency of mutations in *MLO* genes when a representative sample of apple germplasm is considered. In this respect, however, FruitBreedomics re-sequencing data needed first to be validated. Thus, the presence of insertion T-1201 had to be confirmed by Sanger sequencing. The comparison between Sanger sequencing and the FruitBreedomics re-sequencing showed conflicting results. However, the in silico prediction of INDEL is complicated and less reliable than substitutions (Minoche et al. 2011; Robison 2012), therefore, the detection of some inconsistencies was not surprising. Three SNPs (G-1181-A, T-1188-C, and C-1205-T) were found to be always associated to insertion T-1201, suggesting that the insertion is carried only by a specific haplotype. Considering that the FruitBreedomics dataset includes the genome sequences of the 14 individuals from which the large majority of European apple varieties originated (Evans et al. 2011; Bianco et al. 2014), it is interesting that four of them contained insertion T-1201, namely, “McIntosh”, “Jonathan”, “Delicious” and “Priscilla”. It is reasonable to think that the allele present especially in the first three cultivars subsequently spread through their extensive use in breeding worldwide. “Priscilla” has a more limited use in breeding, as it is younger, has been distributed under an incorrect name (Evans et al. 2011), and has probably been used only in the breeding program of Wageningen UR.

Insertion T-1201 was present in 89 individuals, heterozygous in 64, and homozygous in 25. Five of these 89 individuals were mutants. Included as further control of the quality of sequencing, they were all found identical to their individual of origin with regard to the fragment of *MdMLO19* analyzed in this study. However, some differences in the level of resistance were noted, particularly between “Gala” (susceptible) and “Royal Gala” (resistant), as well as between “Delicious” (resistant) and “Red Delicious” (susceptible). These differences are ascribed to the different sources of phenotypic information included in this study.

The CCA showed conflicting results in the two cases considered. This difference can be partially explained by the different origin of the data considered, but not by the fact that the observations were carried out in different geographical areas populated by different *P. leucotricha* strains because *mlo* resistance is known to be broad-spectrum and unaffected by the different strains of the pathogens (Pavan et al. 2010). The contrasting results between the CCAs and the observation that 11 individuals homozygous for insertion T-1201 were susceptible or very susceptible to PM is in contrast with our previous findings in transgenic “Gala”, where the knockdown of *MdMLO19* resulted in a significant reduction of PM susceptibility (Pessina et al. 2016a). The specificity of *MdMLO19* knockdown was tested and confirmed (Pessina et al. 2016a), therefore, the contrast between the two studies cannot be explained by off-target knockdown of other *MLO* genes. However, the present study considered a high number of individuals, whereas the previous one regarded a single cultivar, so it is possible that the specific genetic background of “Gala” is the key to explain the observed discrepancy. The knockdown of *MdMLO19* in other apple cultivars would be necessary to clear this point.

To explain why individuals carrying a homozygous loss-of-function mutation in what is considered a PM S-gene were susceptible to the disease, we here discuss three hypotheses: (1) presence of other mutations in *MdMLO19* that null the effect of insertion T-1201, (2) presence of other S-genes for PM that may substitute the role of *MdMLO19*, and (3) presence of mutations in genes required for defense. The first hypothesis was that the susceptible genotypes could carry other mutations that prevented the formation of the early stop codon. The only mutation found in the FruitBreedomics data that could null the effect of the insertion and cause the regain of the correct reading frame was deletion G-1181 in “Pepino Jaune”, but Sanger sequencing showed that it was not present in any of the considered susceptible individuals. Other mutations could have a similar effect, but they were not found in proximity of insertion T-1201. Although their presence in other parts of *MdMLO19* cannot be excluded, this does not seem likely on the basis of FruitBreedomics data. The second hypothesis contemplates the presence of other S-genes that might interfere with the PM phenotype elicited by *Mdmlo19* recessive mutation. In a previous work, we showed that *MdMLO19* is a susceptibility gene for PM in apple (Pessina et al. 2016a). However, other *MLO* genes might be in play: *MdMLO18* was not considered an S-gene on the basis of the results of a complementation test in *A. thaliana* (Pessina et al. 2016a), but these kinds of test are not as reliable as *in planta* studies. Therefore, the role of *MdMLO18* requires more clarifications. The two other members of apple Clade V, *MdMLO5* and *7*, were not considered because they were not responsive to PM inoculation (Pessina et al. 2014). The choice of excluding *MdMLO5* and *7* from the study was justified by the understanding of the role of *MLO* genes in pathogenesis of that time, but recent results in grapevine revealed that non-responsive genes may have a secondary role (Pessina et al. 2016b). Thus, a role for *MdMLO5* and *7* cannot be excluded. An interesting fact to consider is that *MdMLO19* is the clade V *MLO* genes of apple with the highest basal expression. In cucumber and *Arabidopsis*, it was also observed that the major *MLO* S-genes is the one expressed the most. However, in both species, a minor role in susceptibility for other clade V *MLO* genes was observed, detectable only when the major S-gene was knocked out (Dr. Henk J. Schouten, personal communication; Consonni et al. 2006). In apple, only *MdMLO11* was knocked down together with *MdMLO19*, with no effect on PM resistance (Pessina et al. 2016a), but no information is available for *MdMLO5* and *7.* Therefore, it is possible that these two genes have a redundant effect and partially complement the role of *MdMLO19* in susceptibility. A further option to consider is the presence of other S-genes outside the *MLO* family. The third hypothesis considered the possibility of mutations in genes that are required for an effective response to the infection. The *PEN* genes are a perfect example in this sense, as their knockout in *A. thaliana* restored PM susceptibility in *Atmlo2*-resistant mutant (Consonni et al. 2006). *PEN* genes are well known, but clearly not the only genes involved in pathogenesis, therefore a wider approach will be necessary.

Natural loss-of-function mutations in *MLO* S-genes were found in four species: barley *mlo-11* (Piffanelli et al. 2004), tomato *ol-2* (Bai et al. 2008), pea (Pavan et al. 2011), and cucumber (Berg et al. 2015). If its role will be confirmed, insertion T-1201 in *MdMLO19* would be the fifth. To date, only the germplasms of barley and cucumber were screened for natural *MLO* loss-of-function mutations. Among the around 4100 barley accessions tested, the frequency of spontaneous *mlo* mutations varied between 0.2 and 0.6% (Jørgensen 1992). By contrast, a much higher frequency was observed in cucumber, where a transposon disrupting *CsaMLO8* was detected in 27% of the individuals considered (Berg et al. 2015). The estimate of the frequency of insertion T-1201 in apple *MdMLO19*, based on FruitBreedomics data and Sanger sequencing results, was 9.5% if only homozygous individuals are considered (6 out of 63), 27% if also heterozygous ones are considered (17 out of 63), a result identical to what was observed in cucumber. The presence of insertion T-1201 in apple breeding cultivars can contribute to explain its high frequency.

Alleles of *MdMLO19* carrying insertion T-1201 do not seem to be an immediate source of durable PM resistance in apple, and further studies are required to identify the other genes causing PM susceptibility in apple. The screening of the germplasms of other species might provide more information on the important and yet poorly studied aspect of the frequency of spontaneous *mlo* mutants.

Our results have shown how whole-genome re-sequencing of different individuals of a species, like that on the SNP discovery panel of the FruitBreedomics Axiom 487K array (Bianco et al. 2016), can provide valuable preliminary information for the study of the natural diversity of the germplasm of a species. Furthermore, the screening of re-sequencing databases can lead to the identification of candidates *MLO* S-genes: the presence of homozygous nonsense mutations in specific *MLO* genes of PM-resistant individuals would be an important indication that the gene might act as an S-gene. Finally, this approach could be extended to other diseases and other S-genes.

## Electronic supplementary material

Figure S1 (TIFF 8708 kb)

Figure S2 (TIFF 24647 kb)

ESM 1 (XLSX 643 kb)
