# Supplementary figures and images for: Frequency of a natural truncated allele of MdMLO19 in the germplasm of Malus domestica
Source: Mol Breed. 2017 Jan 4;37(1):7. doi: 10.1007/s11032-016-0610-8 (PMC5214879; doi:10.1007/s11032-016-0610-8)

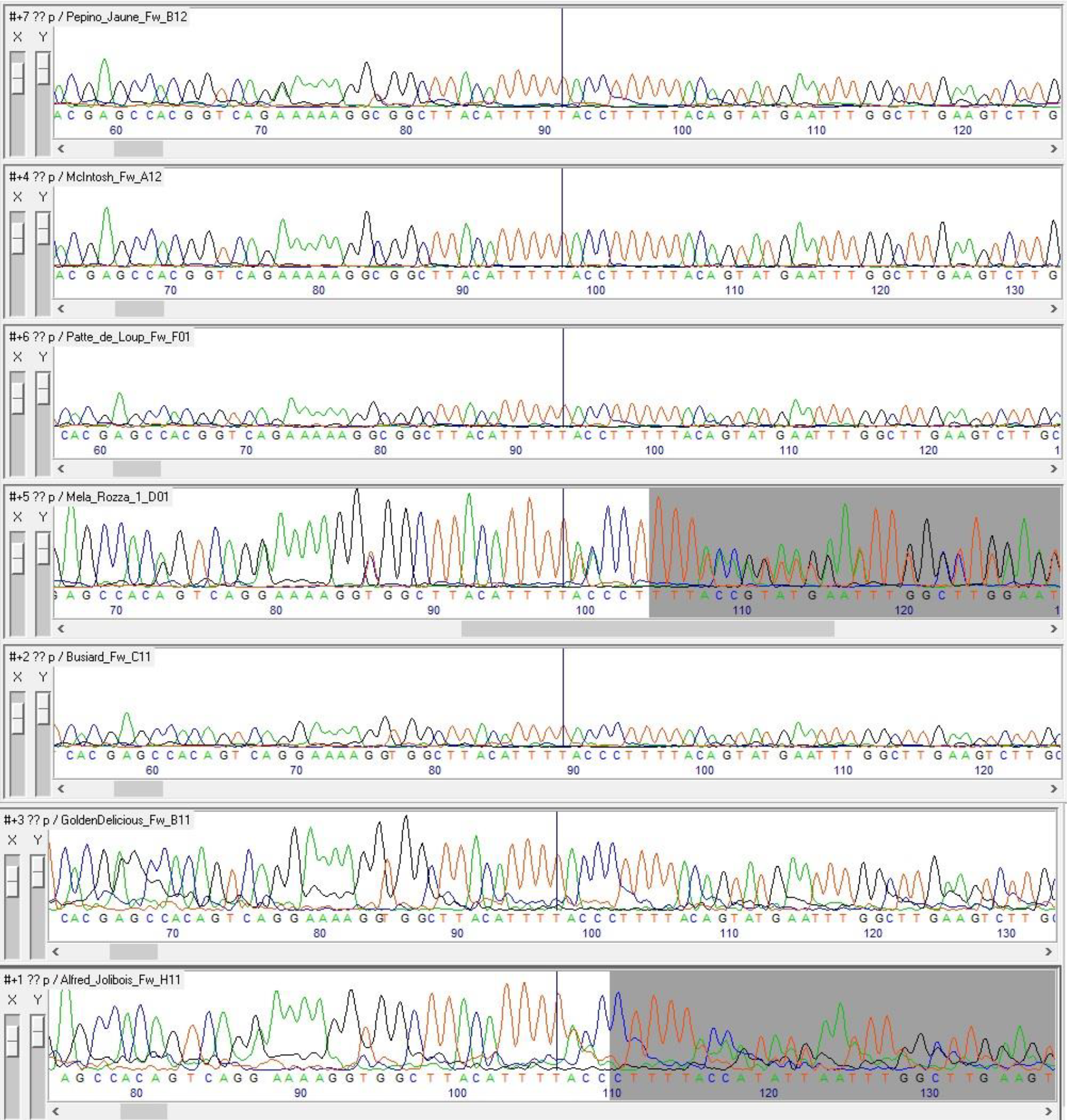

Supplement: Supplementary file 1 — (TIFF 8708 kb) [file 11032_2016_610_MOESM1_ESM.tif]

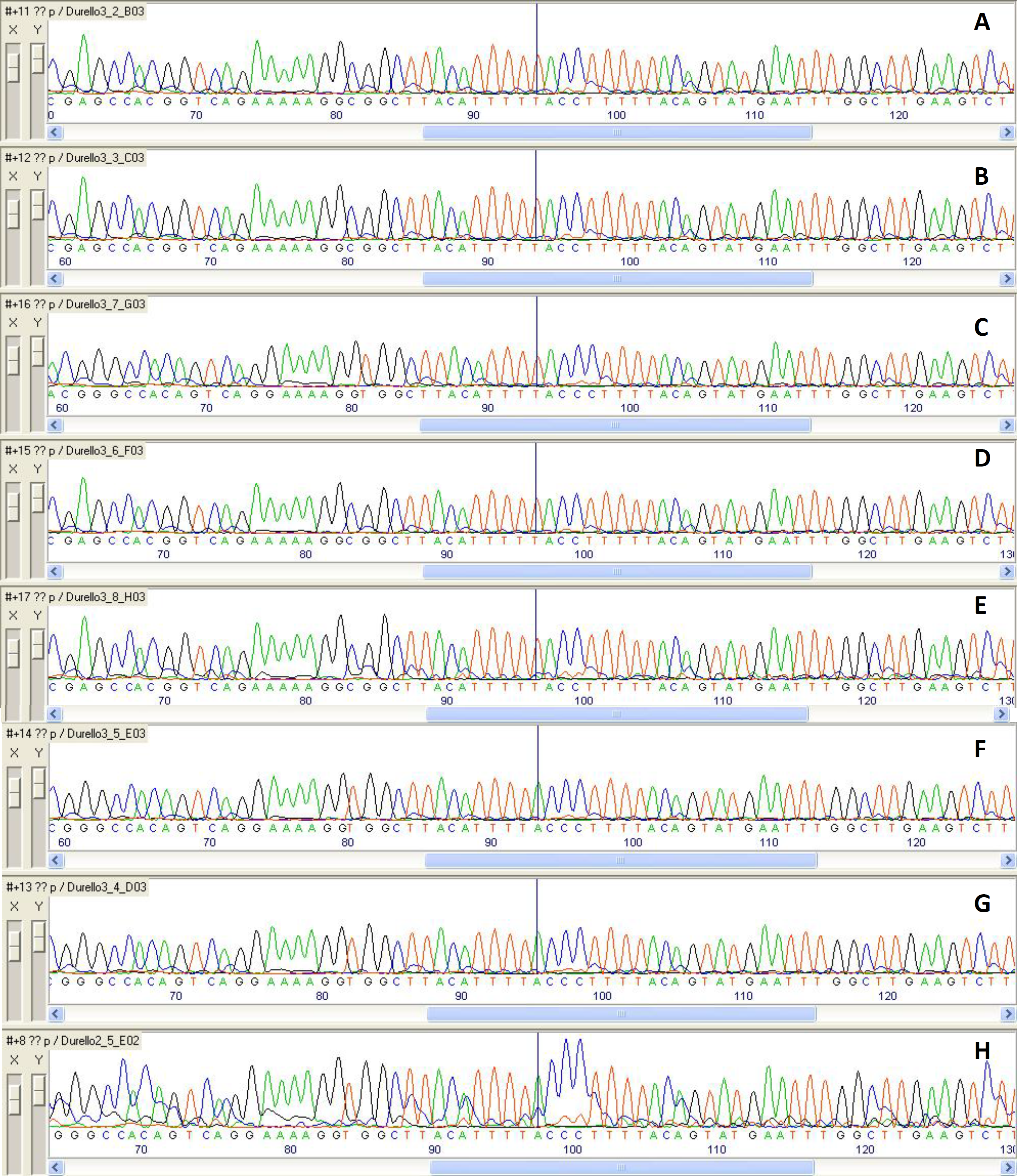

Supplement: Supplementary file 2 — (TIFF 24647 kb) [file 11032_2016_610_MOESM2_ESM.tif]
